# Unveiling therapeutic frontiers: DON/DRP-104 as innovative Plasma kallikrein inhibitors against carcinoma-associated hereditary angioedema shocks - a comprehensive molecular dynamics exploration

**DOI:** 10.1007/s12013-024-01266-0

**Published:** 2024-06-13

**Authors:** Ernest Oduro-Kwateng, Mahmoud E. S. Soliman

**Affiliations:** Molecular Bio-Computation and Drug Design Research Group, School of Health Sciences, University of KwaZulu Natal, Westville Campus, Durban, 4001 South Africa

**Keywords:** Plasma kallikrein, Hereditary Angioedema, Cancer, DRP-104, molecular dynamics

## Abstract

Human plasma kallikrein (PKa) is a member of the serine protease family and serves as a key mediator of the kallikrein-kinin system (KKS), which is known for its regulatory roles in inflammation, vasodilation, blood pressure, and coagulation. Genetic dysregulation of KKS leads to Hereditary Angioedema (HAE), which is characterized by spontaneous, painful swelling in various body regions. Importantly, HAE frequently coexists with various cancers. Despite substantial efforts towards the development of PKa inhibitors for HAE, there remains a need for bifunctional agents addressing both anti-cancer and anti-HAE aspects, especially against carcinoma-associated comorbid HAE conditions. Consequently, we investigated the therapeutic potential of the anti-glutamine prodrug, isopropyl(S)-2-((S)-2-acetamido-3-(1H-indol-3-yl)-propanamido)-6-diazo-5-oxo-hexanoate (DRP-104), and its active form, 6-Diazo-5-oxo-l-norleucine (DON), recognized for their anti-cancer properties, as novel PKa inhibitors. Utilizing structure-based in silico methods, we conducted a comparative analysis with berotralstat, a clinically approved HAE prophylactic, and sebetralstat, an investigational HAE therapeutic agent, in Phase 3 clinical trials. Inhibiting PKa with DON resulted in relatively heightened structural stability, rigidity, restricted protein folding, and solvent-accessible loop exposure, contributing to increased intra-atomic hydrogen bond formation. Conversely, PKa inhibition with DRP-104 induced restricted residue flexibility and significantly disrupted the critical SER195-HIS57 arrangement in the catalytic triad. Both DON and DRP-104, along with the reference drugs, induced strong cooperative intra-residue motion and bidirectional displacement in the PKa architecture. The results revealed favorable binding kinetics of DON/DRP-104, showing thermodynamic profiles that were either superior or comparable to those of the reference drugs. These findings support their consideration for clinical investigations into the management of carcinoma-associated HAE.

## Introduction

**Fig. 1:**
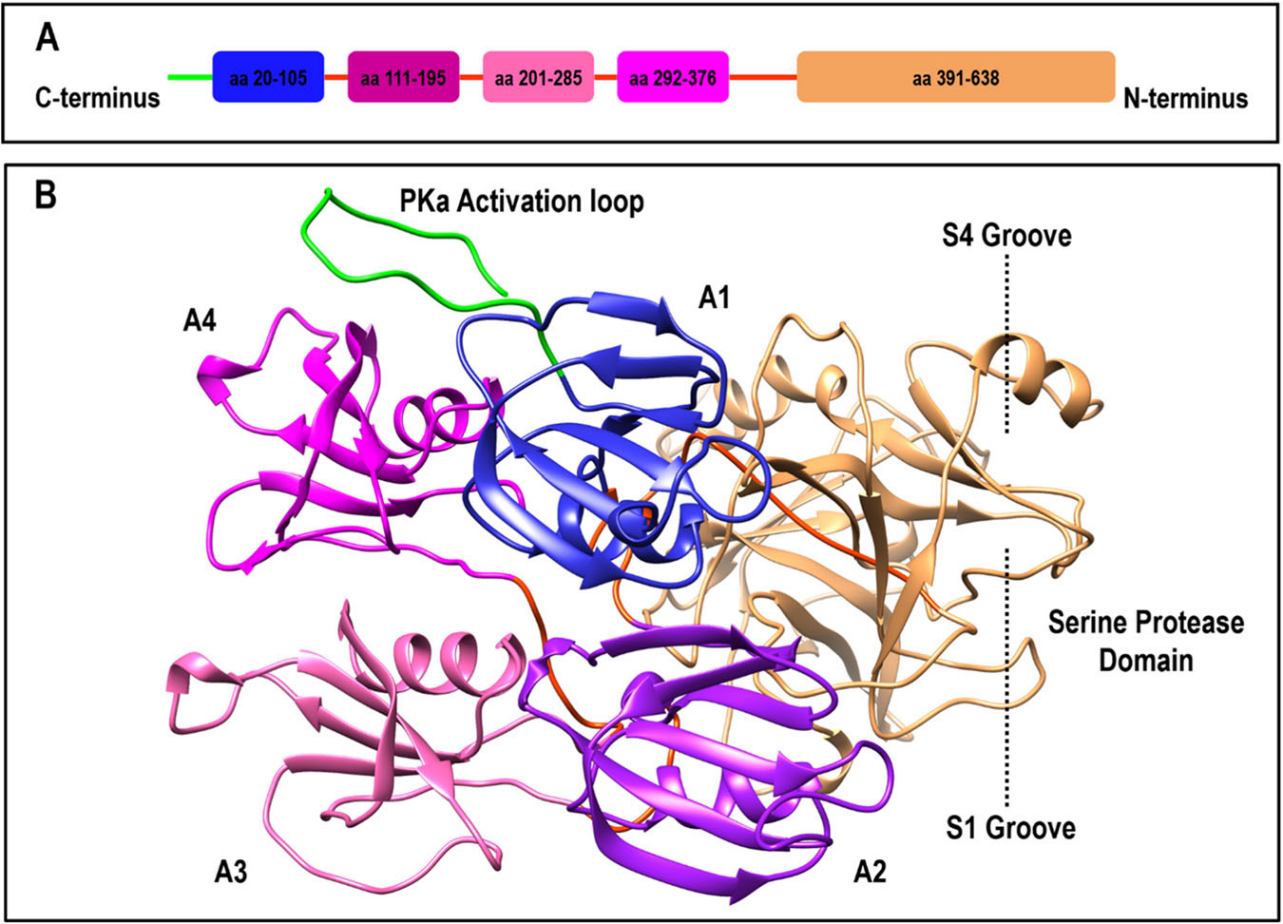
Schematic diagram of (**A**) residue sequence and domain architecture of the four apple domains, A1 (blue), A2 (purple), A3 (pink), and A4 (magenta), and their interconnecting sequence loops (orange) attached to the serine protease domain (sandy brown), and (**B**) 3D crystallized structure of full-length human plasma kallikrein (PDB: 6O1G) obtained by refining missing loops using Modeller program. The PKa activation loop is shown as a pre-sequence (green). Also, the relative positions of the S1 and S4 grooves are shown

Plasma kallikreins (PKa), encoded by KLKB1 on human chromosome 4q35 and synthesized in the liver, belong to the trypsin-like serine protease sub-family with a pivotal role in blood homeostasis, contributing to both proinflammatory and prothrombotic processes [1, 2]. The full-length PKa (619 aa residues) comprises a C-terminal serine protease (SP) domain (ILE391-ALA638) and an N-terminal arrangement of four disc-shaped apple domains, A1 (GLY20-ALA110), A2 (CYS111-GLY200), A3 (CYS201-PRO291), and A4 (CYS292-ARG390), interconnected by disulfide bonds (Fig. 1). The SP domain hosts the catalytic triad (HIS57, ASP102, and SER195) within the active site, governing PKa proteolytic activity. The SP domain comprises two six-stranded β-barrels, creating a distinctive structural motif. The apple domains collectively contribute to substrate recognition, facilitating the recruitment of molecules like high-molecular-weight kininogens (HKs) and factor XII (FXII), as well as substrate-receptor binding. These functions collaboratively modulate the proteolytic activity of PKa [3, 4]. The key function of PKa lies in releasing kinins, specifically bradykinin (Bk) and Lys-bradykinin (Lys-Bk), from high-molecular-weight kininogens (HKs) and low-molecular-weight kininogens (LKs), respectively [5, 6]. The C1 inhibitor (C1-INH) is a crucial regulator in controlling the physiological activity of plasma kallikrein. It rapidly inactivates PKa, preventing the excessive production of bradykinin. This process is essential for maintaining the balance of the kallikrein-kinin system (KKS) and preventing unwanted inflammation or other physiological effects associated with elevated bradykinin levels. Additionally, other circulating inhibitors like α2-macroglobulin and antithrombin III also contribute to the regulation of PKa activity [5, 7]. Upon bradykinin release, the activation of bradykinin receptors (B1R and B2R) initiates intracellular signaling cascades, resulting in increased calcium levels, the release of various mediators, and physiological responses, including inflammation, vasodilation, and pain. In the KKS, PKa is activated from its zymogen, prekallikrein, by factor XIIa (FXIIa). PKa, in turn, contributes to a feedback loop by converting factor XII (FXII) to its active form, FXIIa, further amplifying the activation of the KKS. Beyond its primary role in kinin release, PKa has been reported to mediate other physiological activities such as platelet aggregation, fibrinolysis, complement, pathway and the renin-angiotensin system (RAS) [2, 7].

In addition to antagonizing PKa, C1-INH also antagonizes FXIIa, and an important regulator of the KKS. Hence, diminished levels or abnormal variants of C1-INH due to SERPING1 genetic mutations lead to a pathological state marked by the upregulation of PKa and subsequent elevated bradykinin levels. This condition is identified as Hereditary Angioedema (HAE), a rare autosomal dominant genetic disorder marked by recurring painful swelling in multiple body regions, including the face, extremities, abdomen, genitals, and upper respiratory tract. Alongside Type I and Type II HAE, there is Type III, where individuals exhibit normal C1-INH levels. Management of HAE involves targeting PKa effects, with PKa inhibitors serving as the primary on-demand first-line drug agents [2, 8, 9]. Besides targeting HAE, PKa inhibitors are under development for the treatment of uncontrolled PKa-related conditions, such as diabetic retinopathy, macular edema, and sepsis [5, 10]. Presently, ecallantide [11], lanadelumab [12], and berotralstat [13] are the Food and Drug Administration (FDA)-approved PKa inhibitor drugs for managing Hereditary Angioedema (HAE). Ongoing investigations involve several inhibitors, including ATN-249 (phase I) [14], STAR-0215 (phase 1) [15], KVD824 (phase 2) [9], and KVD900 (phase 3) [7], reflecting a commendable effort to develop newer, safer, and administration-friendly PKa inhibitors. However, with a growing number of reported HAE-associated cancer cases, the quest for PKa inhibitors exhibiting dual functionality—both anti-carcinogenic and anti-angioedema—has become imperative [16–18]. Particularly crucial is the incorporation of prodrug or advanced drug-delivery systems to bolster efficacy while minimizing off-target tissue toxicities. Although the association between Hereditary Angioedema (HAE) and malignancy is not well-defined, certain cancers have been linked to acquired angioedema (AAE), and comorbidity between HAE and cancer has been observed [19, 20]. It is noteworthy that accumulating research suggests a link between the dysregulation of plasma kallikrein (PKa) and the progression of various carcinomas, including lung [21], pancreatic [22], colorectal [23], and breast [24] cancers. In this context, we propose a novel anti-glutamine prodrug, isopropyl(S)-2-((S)-2-acetamido-3-(1H-indol-3-yl)-propanamido)-6-diazo-5-oxo-hexanoate] (DRP-104), as a promising PKa inhibitor. Efficacy studies of DRP-104 against murine lymphoblastic lymphoma (EL4), breast cancer (E0771), colon cancer (MC380), and lung cancer (3LL) cell lines demonstrated clinically accepted tumor regression capabilities, preferential tumor delivery potential, reduced off-target toxicity, and enhanced synergy with anti-PD-1 therapy, accompanied by notable immunologic memory [25]. Our earlier study identified DRP-104 as a potent serine protease inhibitor targeting human TMPRSS2 in the treatment of SARS-CoV-2, warranting further in vitro validation [26].

Our present study utilized in silico methods to explore the structural determinants and dynamic features that govern the inhibitory effects of DRP-104 and its active form, 6-Diazo-5-oxo-l-norleucine (DON), against human plasma kallikrein (PKa). These findings were compared with berotralstat, a clinically prescribed oral prophylactic, and sebetralstat, a Phase 3 oral therapeutic for hereditary angioedema (HAE). Although there are no current clinical reports detailing the half-life or longevity profile of DRP-104 in vivo, pharmacokinetic analyses conducted by Rais et al. [25] demonstrated its remarkable stability in human plasma [25]. These studies revealed that over 90% of DRP-104 remained intact even after 60 min in circulation. These findings were corroborated by Yokoyama et al. [27], who similarly observed the high stability and integrity of DRP-104 in human plasma, alongside its sustained activity [27]. Based on these observations, we hypothesized that DRP-104 possesses the potential to effectively reach plasma kallikrein (PKa) and modulate its functions, thus justifying its selection as an investigational inhibitor. Our findings highlight the anti-PKa potential of both DON and DRP-104 as alternative treatment options for HAE, necessitating further in vitro validation. In view of this, there is a call for drug-design studies focusing on multifunctional prodrug serine protease inhibitors.

## Methodology

### Computational system preparation and binding pocket identification

**Fig. 2:**
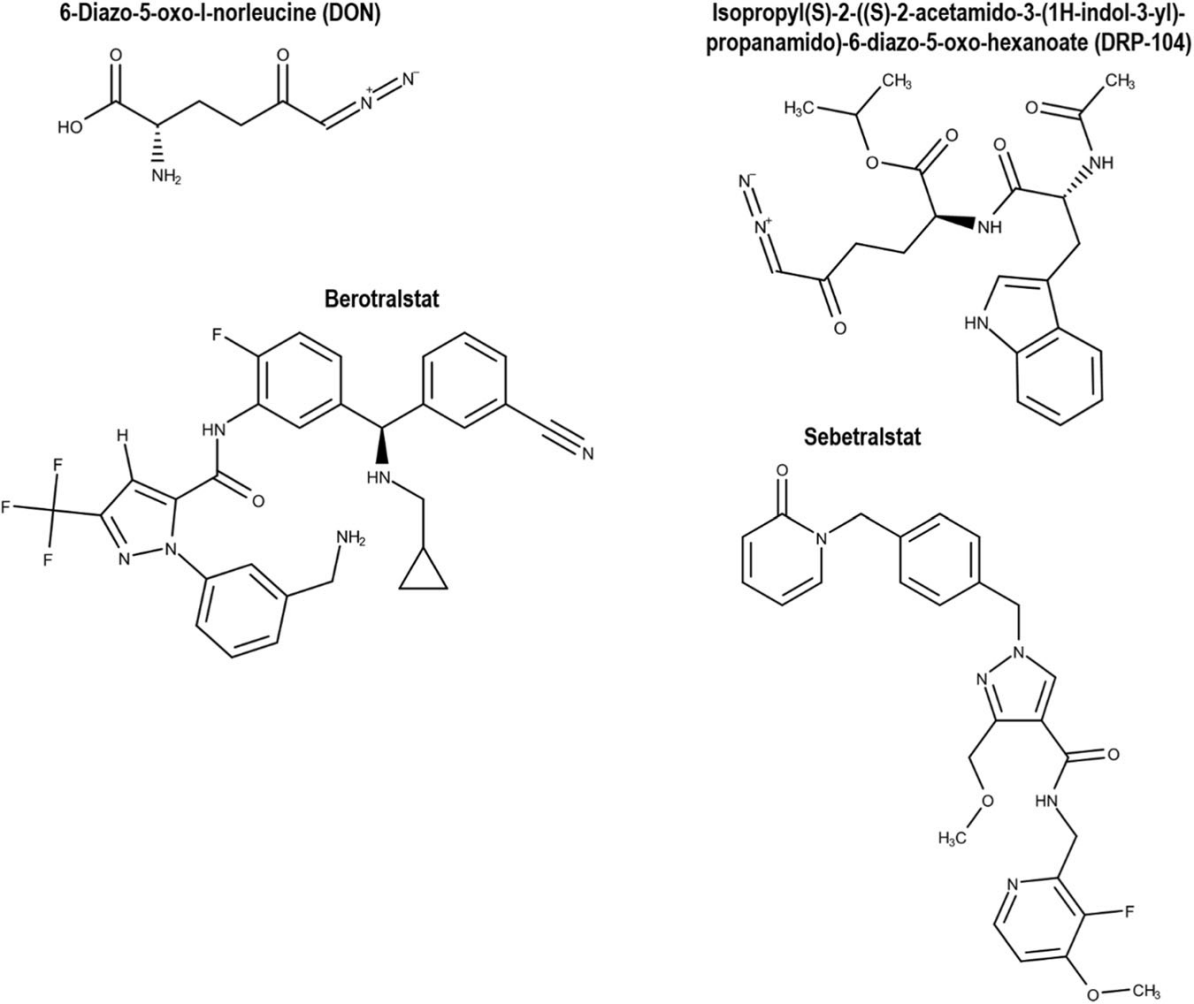
Chemical structures of DON and DRP-104 and the reference drugs, berotralstat and sebetralstat. There is a shared feature of aromatic groups with the exception of DON

The 3D crystal structure of human PKa (PDB: 8A3Q) obtained by x-ray diffraction method at 1.95 Å resolution was retrieved from RCSB Protein Data Bank (https://www.rcsb.org/). The crystallized structure consists of a PKa-SP domain complexed with compound 14w. The protein PDB structure was prepared by removing water, compound 14, and nonstandard molecules. In addition, the protein structure was minimized, hydrogen bonds were added, and protonation states for histidine residues were generated using UCSF Chimera Tools [28, 29]. The active binding pocket of the PKa SP domain was characterized as the interacting binding residue framework for compound 14w [7]. This aligns with findings in other literature, providing confirmation of the designated binding site [1, 3, 6]. The 3D chemical structures of DON, DRP-104, and berotralstat were drawn in MarvinSketch v23.5 (https://chemaxon.com/marvin). Figure 2 shows the 2D structures of the ligands used in this study. The steepest descent algorithm at four steps of the GAFF force field optimization tool on Avogadro v1.2.0 workspace was used to minimize the thermal energies of each ligand [30]. The ligands were then optimized on Molegro Molecular Viewer v7.0.0 (MMV), and bond angles and hybridization states were corrected where necessary [31]. Further minimization of the ligands was performed at 100 steepest descent steps, 0.02 steepest descent steps size (Å), 10 conjugate gradient steps, and 0.02 conjugate gradient step size (Å) of 10 update intervals on UCSF Chimera Tools. UCSF Chimera Tools was used to optimize the atomic coordinates of the ligands, and Gasteiger charges were added using ANTECHAMBER tool [32].

### Molecular docking calculations

AutoDock Vina was employed to carry out docking calculations using the Lamarckian Genetic Algorithm on UCSF Chimera v1.17.1 [31]. AutoDock Vina performs docking calculations using an advanced gradient optimization technique and incorporates multithreading to enhance both speed and precision in the clustering of grid map results. Notable for its efficiency and accuracy, AutoDock Vina stands out as a molecular docking program, consistently surpassing the performance of other widely adopted docking software programs [33, 34]. The molecular docking of the systems was carried out, focusing on the recommended active site to enhance precision and reduce potential false positive interactions with ligands. The Autodock Tools interface was utilized to specify the grid volume within the active site of human plasma kallikrein, employing 5 Å spacing in the *x*, *y*, and *z* dimensions. The grid center was set at coordinates (20.2365, 179.5460, 115.0500), and the dimensions were defined as (17.9130 × 24.0958 × 21.0737), respectively [31] The docking poses were observed within the Chimera workspace, and the reported docking scores were determined based on the lowest negative values relative to the optimal mode. This thorough approach ensured a comprehensive and precise evaluation of our molecular docking results.

### Molecular dynamics (MD) simulation

UCSF Chimera Tools were employed for pre-molecular dynamics (MD) preparations of PKa and ligands. MD simulations for free and ligated PKa complexes utilized the Particle Mesh Ewald Molecular Dynamics (PMEMD) Compute Unified Device Architecture (CUDA) on a single graphic processing unit (GPU) integrated into the AMBER 18 package [35, 36]. To add partial charges to inhibitors, the ANTECHAMBER protocol applied restrained electrostatic potential (RESP) and GENERAL AMBER Force Field (GAFF) procedures, while the FF14SB AMBER force field parameterized proteins. Solvation and neutralization were accomplished by the LEAP module, incorporating hydrogen atoms, sodium ions, and chloride counter ions. Systems were solvated in an orthorhombic TIP3P box with 10 Å water molecules. The pdb4amber command modified PKa protein system topologies before executing the LEAP protocol. Partial minimization (2500 steps) with a 500 kcal/mol restraint potential and full minimization (5000 steps) without conjugate energy restraint were conducted. Furthermore, a heating process from 0 to 300 K for 50 ps occurred in a canonical ensemble (NVT). This employed a Langevin thermostat, a 1 ps random collision, and a harmonic potential restraint of 10 kcal/mol Å. Hydrogen bond constraints were managed by the SHAKE algorithm under a 1 bar pressure from Barendsen-Barostat. Systems underwent 200 ns MD simulations in an isothermal-isobaric (NPT) ensemble, with a time scale of 2 fs, a Langevin thermostat at 300 K, and a constant pressure of 1 bar. Coordinate data were saved every 1 ps and analyzed using the CPTRAJ and PTRAJ modules of AMBER18 GPU [37]. Post-MD analyses covered root mean square deviation (RMSD), root mean square fluctuation (RMSF), radius of gyration (ROG), solvent-accessible surface area (SASA), Dynamic Cross-Correlation Matrix (DCCM), Principal Component Analysis (PCA), and hydrogen bonding calculations. Visualization and structural analyses utilized VMD and UCSF Chimera graphical software packages, whereas Origin software was employed for generating graphs and data plots [29, 31].

### Receptor—ligand interaction analysis

For visualization of binding modes and analysis of non-covalent residue molecular interactions, Biovia Discovery Studio Visualizer v21.1.0.20298 was utilized [31, 38]. This tool was instrumental in examining the bond types and molecular interactions between PKa and the ligands.

### Binding free energy (BFE) computations

The Molecular Mechanics/Generalized-Born Surface Area (MM/GBSA) protocol was employed to estimate the free energy of binding (BFE) between PKa and ligands within the bound complexes. MM/GBSA is widely utilized in structure-based drug design and molecular modeling studies for predicting and analyzing binding affinities, evaluating binding poses, and comprehending the energetics of molecular interactions within a biological system [39]. This method offers a more detailed description of binding free energies compared to scoring functions used in docking studies. The MM/GBSA approach integrates molecular mechanics calculations, which employ classical force fields to describe molecular interactions, with the Generalized-Born (GB) dielectric continuum solvent model, and surface area (SA) terms to estimate the BFE [40]. Molecular mechanics calculations encompass internal energy, van der Waals interactions, electrostatic interactions, and other molecular forces. The GB model estimates polar solvation-free energy by considering the Born radii of atoms and their pairwise interactions. In contrast, the SA method quantifies the reduction in hydrophobic interactions upon binding through the computation of the buried surface area (BSA) during complex formation, correlating the nonpolar solvation energy correlates with the surface area of the protein–ligand interface using a water probe radius of 1.4 Å and surface tension constant (γ) set at 0.0072 kcal/mol Å^2^. BFE calculations were estimated using 200000 complex frames from the 200 ns trajectories. The formula for BFE (∆G) computation is given as:

$${\Delta G}_{{bind}}={G}_{{complex}}-{G}_{{receptor}}-{G}_{{ligand}}$$

$${\Delta G}_{{bind}}={E}_{{gas}}+{G}_{{sol}}-T\Delta S$$

Where:

$${E}_{{gas}}={E}_{\mathrm{int}}+{E}_{{vdw}}+{E}_{{ele}}$$

$${G}_{{sol}}={G}_{{GB}}+{G}_{{SA}}$$

$${G}_{{SA}}=\gamma {SASA}$$

Here, ∆*G*_*bind*_ represents the gas-phase summation, *E*_*gas*_ is the gas-phase energy, *G*_*sol*_ is the free solvation energy, *T*∆*S* is the total interaction entropy, *E*_*int*_ is the internal energy, *E*_*ele*_ is the coulomb energy, and *E*_*vdw*_ is van der Waals energy. *E*_*gas*_ was computed from the AMBER FF14SB forcefield, and *G*_*sol*_ was calculated from the energy contributions of polar and non-polar states.

### Per-residue energy decomposition (PRED) analysis

PRED Analysis using the MM/GBSA method in AMBER 18 was utilized to calculate the energy contribution of each residue to the total BFE of the PKa-bound systems, considering 200000 frames [31].

## Results and discussion

### Molecular docking calculations of PKa inhibitors: enhanced binding affinity via docking poses at S1/S4 Grooves

**Fig. 3:**
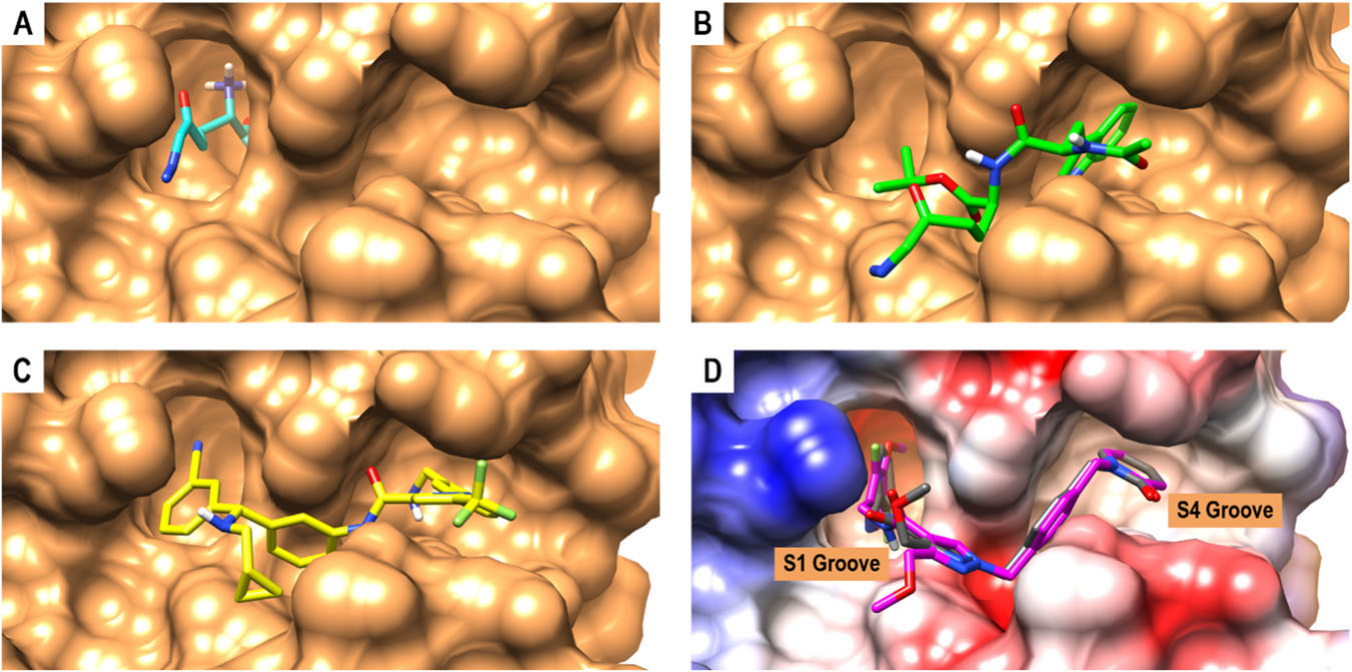
3D docking complex of PKa (sandy brown) and (**A**) DON (cyan), (**B**) DRP-104 (green), (**C**) berotralstat (yellow), and (**D**) sebetralstat (magenta) aligned with crystallized sebetralstat (dark gray) (PDB: 8A3Q). The relative positions of the S1 and S4 grooves are shown along with the columbic surface coloring of PKa-SP domain

Targeting the serine protease (SP) domain of human plasma kallikrein (PKa) has been identified as a viable approach for drug design [2, 9]. The SP domain is divided into distinct sub-domains (S1-S4/S1’) based on their positions along the amino or carboxyl terminus and their respective amino acid cleavage sites of substrate P1-P4 or P1’-P4’. Successful drug design studies emphasize the importance of potent PKa inhibitor moieties that effectively co-bind to both the S1 and S4 grooves [6, 7, 13]. Our docking study validated this observation by revealing varying binding affinities, binding modes, and interacting residue landmarks among the inhibitors (Fig. 3). Sebetralstat had the highest binding affinity (−10.2 kcal/mol), followed by berotralstat (−8.3 kcal/mol), DRP-104 (−7.7 kcal/mol) and DON (−5.8 kcal/mol) (Table 1). The S1 groove forms a deep enclosed cylindrical pocket, opposite to the relatively open and shallow S4 groove, which is completely neutral (Fig. 3D). Notably, the SP domain of PKa is generally neutral, with a few regions having a hydrophobic (electronegative) potential. DON was completely submerged in the S1 groove with its acetic acid group touching the bottom, and its 1‐diazopropan‐2‐one group pointed towards the opening of the group (Fig. 3A). DRP-104 had no contact with the S1 groove. Instead, the indole group engages deeply with the S4 groove (Fig. 3B). Furthermore, the benzylamine group of berotralstat engaged deeply with the S4 group, whereas its benzonitrile group only hovered over the opening of the S1 groove (Fig. 3C). Finally, the 1,2‐dihydropyridin‐2‐one and 3‐fluoro‐4‐methoxypyridine groups of sebetralstat engaged deeply with the S4 and S1 grooves, respectively (Fig. 3D).

**Table 1:** Protein–ligand docking scores and interacting residues of DON and DRP-104 with the SP domain of human plasma kallikrein, compared to berotralstat and sebetralstat.

Inhibitor of plasma kallikrein	Docking score (kcal/mol)	Interacting residues within 5 Å	Number of Hydrogen bonds
DON	−5.8	*HIS57*, VAL138, ASP189, **ALA190,** **CYS191,** **LYS192**, GLY193, ASP194, *SER195*, THR213, **SER214,** **TRP215**, GLY216, GLY219, CYS220, ALA221, PRO225, **GLY226,** **VAL227,** **TYR228**	8
DRP-104 (Sirpiglenastat)	−7.7	*HIS57*, ASP60, TYR94, SER97, GLU98, **GLY99,** *ASP102*, TYR172, TYR174, LYS175, ILE176, MET180, LYS192, *SER195*, SER214, **TRP215**, GLY216, VAL227	2
Berotralstat	−8.3	*HIS57*, ASP60, TYR94, **SER97**, GLU98, **GLY99**, ASN100, *ASP102*, **TYR174**, LYS175, ILE176, MET180, CYS191, LYS192, GLY193, *SER195*, SER214, TRP215, **GLY216**, GLY219, CYS220, VAL227	4
Sebetralstat	−10.2	***HIS57***, ASP60, TYR94, SER97, GLU98, **GLY99**, ASN100, *ASP102*, TYR172, TYR174, LYS175, ILE176, THR177, MET180, **ASP189**, ALA190, CYS191, **LYS192**, GLY193, ASP194, *SER195*, THR213, **SER214**, TRP215, GLY216, GLY219, CYS220, GLY226, VAL227, TYR228	5

Hydrogen-bond-forming interacting residues are highlighted

Residues involved in hydrogen bonding have been boldened and residues of the catalytic triad have been italicized

We validated our docking protocol by superimposing the computed 3D structure of sebetralstat with its crystallized structure (PDB: 8A3Q). This comparison revealed a complete alignment and a similar residue interaction framework between the two structures (Fig. 3D). The binding poses suggest that effective binding to both the S1 and S4 grooves enhances the inhibitory potential of the PKa inhibitor. These docking results offer valuable insights for the development of chemical scaffolds for the treatment of hereditary angioedema (HAE) and related carcinomas. Additionally, this study highlights the superior binding potential of sebetralstat over berotralstat, whereas also underscoring the inhibitory potentials of DRP-104 and DON. These findings warrant further in vitro validation and potential clinical use of DRP-104/DON against PKa.

### Ligand-induced thermodynamic features of human plasma kallikrein

**Fig. 4:**
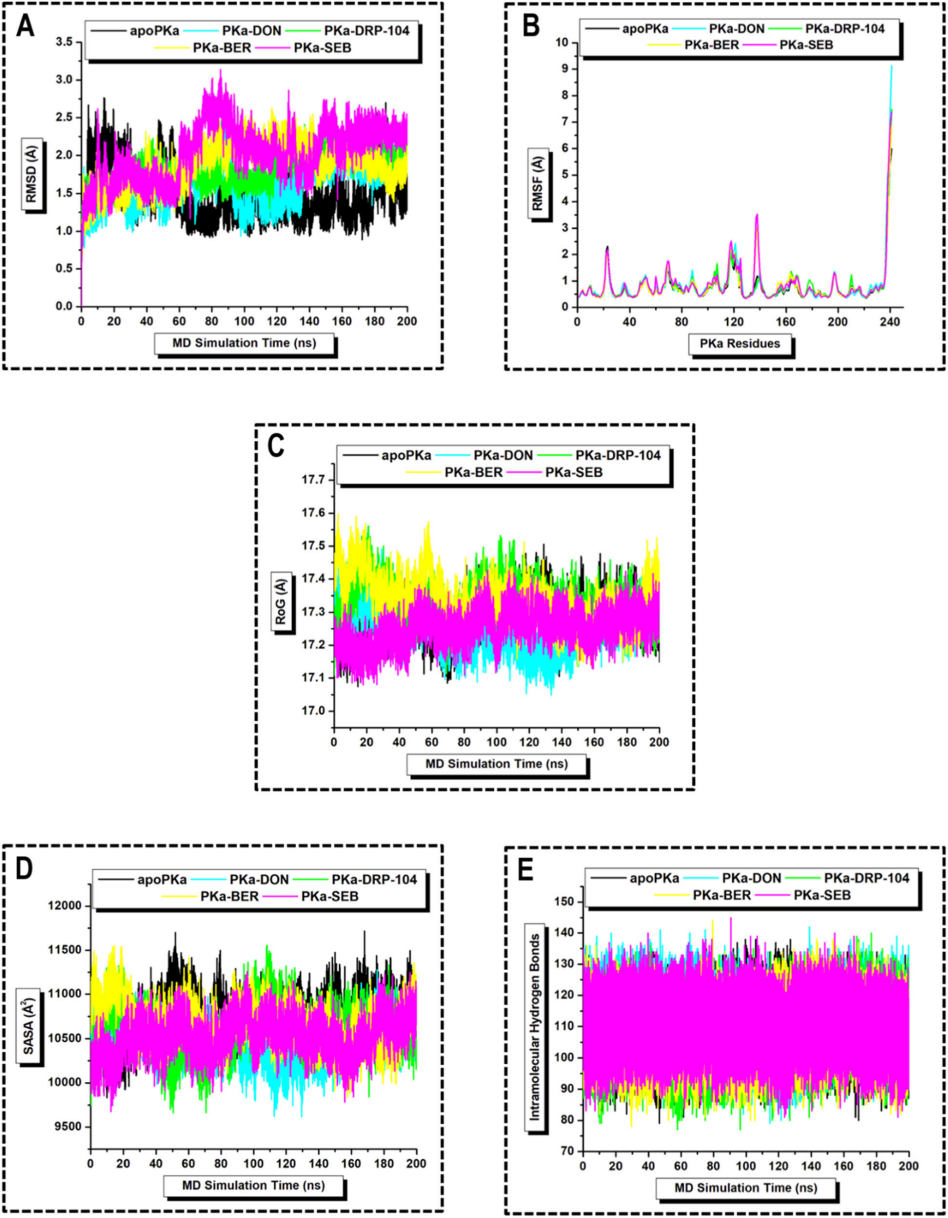
Graphical representation of (**A**) RMSD, (**B**) RMSF, (**C**) RoG, (**D**) SASA, and **(E**) intramolecular hydrogen bonds for the backbone c-α atoms of unligated apoPKa (black), DON- (cyan), DRP-104- (green), berotralstat- (yellow), and sebetralstat- (magenta) PKa ligated complex systems over 200 ns MD simulation

Molecular dynamics simulations provide crucial insights into the dynamic changes of a protein–ligand complex over time, especially in the context of conformational alterations occurring in a biological enzyme when bound to a substrate. These observations are pivotal for advancing structure-based drug design [41, 42]. In this study, we conducted comprehensive post-MD analyses, including RMSD, RMSF, RoG, SASA, PCA, DCCM, and hydrogen bonding calculations, to elucidate the mechanistic features of PKa upon binding DON and DRP-104, in comparison to berotralstat and sebetralstat [29, 31]. Root mean square deviation (RMSD) was employed to assess the structural stability of the backbone c-α atoms of all PKa systems, as they attained convergence or deviation, throughout the 200 ns MD simulations. A lower RMSD value signifies a higher structural stability, whereas a higher value suggests an increased deviation. The average c-α RMSD value of apoPKa, PKa-DON, PKa-DRP-104, PKa-BER, and PKa-SEB was 1.48 ± 0.32 Å, 1.64 ± 0.28 Å, 1.82 ± 0.22 Å, 1.87 ± 0.29 Å, and 2.03 ± 0.34 Å respectively, which are within reference values. All PKa systems underwent significant structural fluctuations over the entire MD period, with apo-DRP-104 and apo-SEB exhibiting marginalized and perturbed fluctuations, respectively (Fig. 4A). Results from RMSD computations suggest that PKa is in its most structurally stable state when unligated than when ligated, and it is more stable when bound to DON than DRP-104 and the reference drugs. Comparatively, sebetralstat decreased the stability of PKa by the highest margin and recorded the highest RMSD value of 3.14 Å at ~85 ns. Root mean square fluctuation (RMSF) was used to ascertain the degree of PKa residues in relation to their local position as they underwent the 200 ns MD simulation when unligated and ligated. A higher RMSF value is indicative of magnified flexibility with decreased kinetic stability, whereas a lower RMSF value indicates restricted flexibility and increased kinetic stability. The average c-α RMSF value of apoPKa, PKa-DON, PKa-DRP-104, PKa-BER, and PKa-SEB was 0.77 ± 0.66 Å, 0.84 ± 0.89 Å, 0.82 ± 0.74 Å, 0.81 ± 0.73 Å, and 0.87 ± 0.85 Å, respectively. We can infer from our RMSF results that the binding of PKa with an inhibitor does not significantly alter the kinetic stability of its residues and maintains its overall residue flexibility. The binding of sebetralstat to PKa increases its flexibility and binding potential compared to DON/DRP-104 and berotralstat. Generally, residues 237–241 experienced the highest degree of flexibility than residues 22–23 in both the apo and ligated PKa systems (Fig. 4B). Thus, these residues may not be important for PKa drug design studies. In addition, residues 116–125 experienced a high degree of flexibility in PKa-DRP-104, PKa-BER, PKa-DON, and PKa-SEB, in increasing order. In contrast, residues 136–139 experienced restricted residue motions in both PKa-DON and PKa-DRP-104, and they experienced loose residue motion in PKa-SEB compared to PKa-BER. Furthermore, radius of gyration (RoG) computation was used to measure the degree of deviation of the c-α atoms around the center of gravity to ascertain their compactness over the 200 ns MD simulation. A high RoG value indicates a less compact protein system, whereas a low RoG value indicates a more compact protein system. The average c-α RoG value of apoPKa, PKa-DON, PKa-DRP-104, PKa-BER, and PKa-SEB was 17.30 ± 0.06 Å, 17.26 ± 0.07 Å, 17.35 ± 0.06 Å, 17.35 ± 0.07 Å, and 17.30 ± 0.07 Å, respectively. We observed insignificant variations in the degree of compactness between the five systems. However, the PKa residues experienced the highest degree of compactness when bound to berotralstat compared to the other inhibitors (Fig. 4C). We can infer that PKa assumes the most compact conformation when bound to DON as opposed to when bound to either of the reference drugs, which may be due to the binding of only the S1 groove in the case of DON in comparison to the binding of both S1 and S4 grooves in the case of the reference drugs (Fig. 3).

### Protein–ligand complex exposure and intramolecular hydrogen bonding: DON-inducing conformation stability in human plasma kallikrein

The solvent-accessible surface area (SASA) technique was used to measure the relative degree of exposure of the hydrophobic residues of PKa to the enclosed solvent in proportion to the degree of packing of the hydrophobic residues towards the protein core. SASA is very useful for ascertaining the solvent-like nature of a protein in response to binding to an inhibitor, as the degree of its folding/unfolding profile over a simulated period of time [43]. The mean c-α SASA value of apoPKa, PKa-DON, PKa-DRP-104, PKa-BER, and PKa-SEB was 10762.25 ± 250.41 Å^2^, 10593.45 ± 257.76 Å^2^, 10701.43 ± 242.54 Å^2^, 10621.03 ± 262.55 Å^2^, and 10625.84 ± 266.44 Å^2^, respectively. Throughout the 200 ns MD simulation period, all PKa systems exhibited relatively similar degrees of hydrophobic surface seclusion, with variations within the error margin (Fig. 4D). However, despite these minor variations, the SASA computations suggest that unligated PKa generally has a slightly higher unfolding potential than ligated PKa. Specifically, DON induced minimal surface exposure in its PKa complex, contributing to hydrophobic stability, whereas DRP-104 resulted in a slightly higher degree of surface exposure in its PKa complex. Similarly, berotralstat provided favorable hydrophobic stability compared with sebetralstat, albeit within the error margin. Thus, the subtle differences observed were statistically significant within the measurement error margins. The consistent hydrophobic surface seclusion observed across all the PKa systems may be attributed to several factors. First, despite the presence of different inhibitors, the overall molecular structure of PKa remains unchanged. Second, the inhibitors may have exerted limited influence on the overall structure and folding dynamics of PKa. Additionally, the relatively short simulation period of 200 ns may not have been sufficient to capture pronounced variations in hydrophobic surface seclusion, necessitating future studies to consider longer simulation times to observe more pronounced effects of these inhibitors on PKa folding dynamics [44].

In addition, intramolecular hydrogen bonding (hb) calculations were performed to gain insights into the conformational dynamics and overall stability of PKa over the 200 ns MD simulations and how these changes differ when bound to DON/DRP-104 in comparison to the reference drugs. This is because intramolecular hbs govern the architectural integrity and functionality of biological enzymes [45]. The mean c-α intramolecular hb value of apoPKa, PKa-DON, PKa-DRP-104, PKa-BER, and PKa SEB was 109.26 ± 6.80, 111.06 ± 6.99, 107.60 ± 7.03, 108.14 ± 6.84, and 110.32 ± 6.78, respectively. Our findings suggest insignificant variations in the number of hydrogen bonds formed over time in the apo versus ligated PKa systems (Fig. 4E). There was an increase in HBs in PKa when bound to DON and sebetralstat and a decrease in HBs in PKa when bound to DRP-104 and berotralstat, as a measure of increased folding and deceased folding potentials, respectively. These findings are consistent with the results from SASA which underscores the high conformational stability in PKa-DON than PKa–sebetralstat complex. Thus, all the inhibitors used in our study formed stable complexes with human plasma kallikrein throughout the MD simulation period. Therefore, both DON and DRP-104 are ideal candidates for PKa and require further clinical validation.

### Assessment of collective residue movements and relative dispersion in plasma kallikrein inhibition

**Fig. 5:**
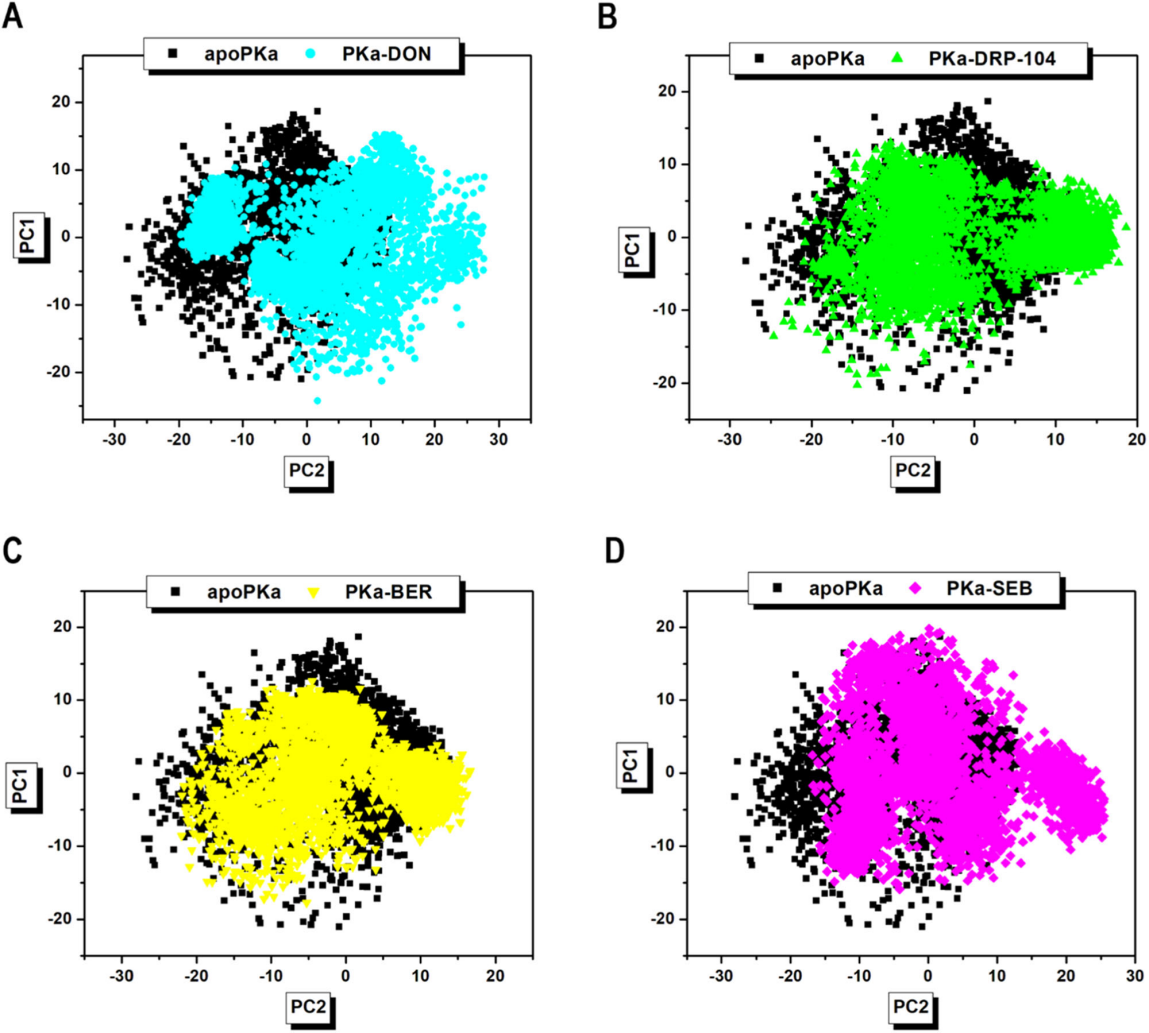
Graphical representation of PCA analysis showing the relative degree of c-α residue displacements in unligated apoPKa (black) versus (**A**) DON- (cyan), (**B**) DRP-104- (green), (**C**) berotralstat- (yellow), and (**D**) sebetralstat- (magenta) PKa ligated complex systems along PC1 and PC2 over 200 ns MD simulation

Atomic motion assessment tools such as the dynamic cross-correlation matrix (DCCM) and principal component analysis (PCA) were used to assess the relative movements and displacements, respectively, of c-α intra-atomic residues of plasma kallikrein (PKa) in response to the induced fit effect of DON/DRP-104 in relation to the reference drugs. These computations were made from MD snapshot trajectories obtained from the 200 ns MD simulation [46]. PCA was used to provide insight into the allosteric behavior of the inhibitors by capturing the essential motions and conformational changes that occur in PKa. PCA calculations used the first and second principal components, PC1 and PC2, in the eigendecomposition of the covariance matrix. PC1 and PC2 represent the first- and second-most significant modes of motion along eigenvectors 1 (ev1) and 2 (ev2), respectively. The results from our PCA analysis showed a dense cluster of MD snapshot trajectories among all five systems. This indicates that all systems reached a stable conformational state during the simulation period. In addition, the correlated motions of the atomic coordinates were bidirectional along both principal components (Fig. 5). Furthermore, atomic displacements were more pronounced in the PKa-DON complex than in the PKa–sebetralstat complex but were less pronounced in the PKa–berotralstat complex than in the PKa-DRP-104 complex. Overall, PKa-DON exhibited the largest degree of atomic displacement.

**Fig. 6:**
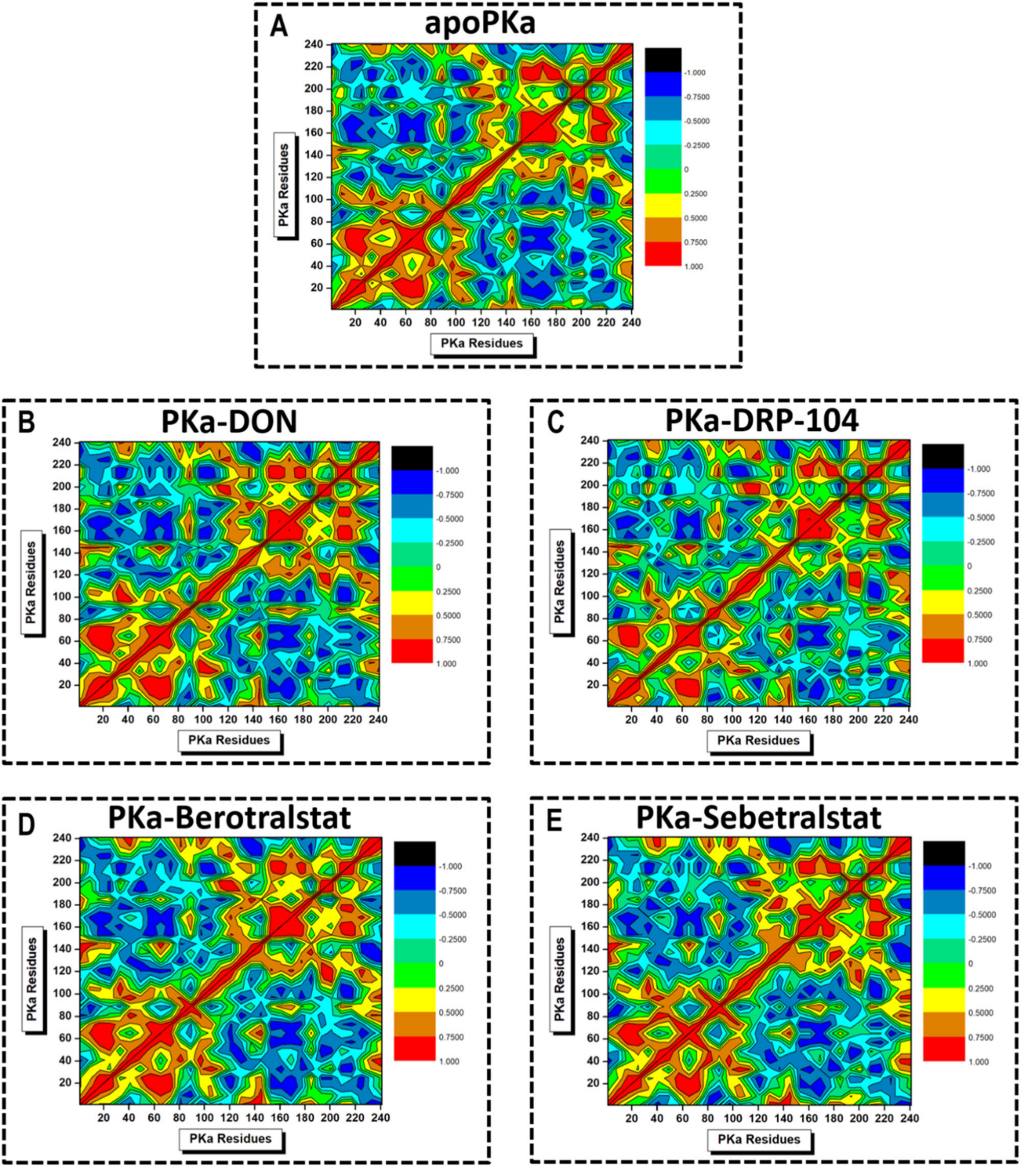
Graphical representation of DCCM analysis showing the relative degree of c-α residue correlated motion in (**A**) apoPKa, (**B**) DON-, (**C**) DRP-104-, (**D**) berotralstat-, and (**E**) sebetralstat-PKa ligated complex systems along PC1 and PC2 over 200 ns MD simulation. The colored contours on the right show the increasing strength of the correlated motion from black to red

DCCM was used to understand the catalytic functions of the inhibitors by assessing the degree of c-α intra-atomic motion and whether they moved together or in opposite directions over the simulated time scale. DCCM can be interpreted within a scale of +1 (strongly positive) to 0 (no correlation) to −1 (strongly negative) motions, represented by red to yellow, light green to green, and cyan to black contours, respectively [29]. Positive correlations indicate regions involved in cooperative motions related to function, whereas negative correlations suggest structural conformational changes. The results of the DCCM analysis revealed that various regions in PKa exhibited a high degree of synchronized motion in PKa–sebetralstat, apoPKa, PKa-DON, PKa-DRP-104, and PKa–berotralstat, in decreasing order (Fig. 6). It is noteworthy that no residue groups in any of the systems underwent strong anticorrelated motions. These findings, together with those from PCA and DCCM, underscore the favorable structural integrity, rigidity, and stability of human plasma kallikrein, even when bound to an inhibitor. This further supports the notion that both DON and DRP-104 are promising candidates for anti-PKa drugs.

### Distance metric analysis of the catalytic triad residues in human plasma kallikrein inhibition

The catalytic mechanism of serine proteases involves a series of steps. Initially, the substrate binds to the protease active site. Subsequently, the catalytic serine residue initiates nucleophilic attack on the carbonyl carbon of the substrate, resulting in the formation of a tetrahedral intermediate. This intermediate then undergoes breakdown to form an acyl-enzyme intermediate, ultimately leading to release of the cleaved product. The catalytic triad, which consists of serine (SER195), histidine (HIS57), and aspartic acid (ASP102) residues, plays a crucial role in coordinating these steps. The histidine residue acts as a general base, activating the serine nucleophile, whereas the aspartic acid residue stabilizes the active form of histidine. Furthermore, the oxyanion hole within the active site is vital for stabilizing the tetrahedral intermediate, thereby contributing to efficient catalysis [47, 48]. In view of this, we hypothesized that disrupting the spatial proximity between SER195 and HIS57 and eventually HIS57 and ASP102 is ideal for curtailing the functionality of the catalytic triad, thereby impairing the catalytic function of human plasma kallikrein (PKa). Therefore, we employed distance metric analysis to measure the distances between residues of the catalytic triad before and after the 200 ns MD simulation period to ascertain the degree to which DON and DRP-104 influenced the catalytic triad machinery in relation to the reference drugs, berotralstat and sebetralstat (Table 2). In this case, a higher distance metric may indicate reduced functionality of catalysis and vice versa. The SER195-HIS57 distance increased upon binding to all inhibitors. Comparatively, the highest increment was observed in PKa-DON and PKa-DRP-104 complexes before and after MD, respectively. However, berotralstat reduced the SER195-HIS57 distance both before and after MD. Hypothetically, this may imply that inhibition of PKa with berotralstat may still favor the catalytic reaction, whereas inhibition with DRP-104 may abrogate such a reaction. In contrast, the HIS57-ASP102 distance showed variable changes, among the inhibitors. The HIS57-ASP102 distance increased most significantly in the PKa-DRP-104 and PKa–sebetralstat complexes before and after MD simulation, respectively. Interestingly, the highest decrement was observed in PKa–sebetralstat complex before MD simulation, and in both PKa-DON and PKa-DRP-104 complexes after MD simulation (Table 2). Notably, sebetralstat induced an increase in both distance metrics only after MD, underscoring that it is an ideal inhibitor to abrogate catalysis in PKa. In addition, both distance metrics decreased in unligated PKa after MD simulation, assuming a stable conformation. Thus, enzymatically active PKa is most likely to undergo catalysis as opposed to when inactive. This further confirms the need to abrogate the functionality of the catalytic triad machinery using an ideal inhibitor in the fight against PKa-related disease conditions.

**Table 2:** Intra-atomic distance metric analysis between the catalytic triad residues (SER195-HIS57-ASP102) in unligated and ligated human plasma kallikrein (PKa) systems before and after the 200 ns MD simulation

Plasma kallikrein system	Distance (Å) measured between the catalytic triad residues			
	Before MD (0 ns)		After MD (200 ns)	
	SER195 HG -HIS57 ND2	HIS57 ND1-ASP102 OD2	SER195 HG -HIS57 ND2	HIS57 ND1-ASP102 OD2
apo	3.532	2.794	3.428	2.743
apo-DON	3.885	2.793	3.456	2.723
apo-DRP-104	3.480	2.808	4.147	2.723
apo-Berotralstat	3.168	2.753	3.421	2.768
apo-Sebetralstat	3.407	2.687	3.617	2.972

### Free energy of binding analyses in human plasma kallikrein inhibition

The molecular behavior of a protein–ligand complex is governed by intermolecular energy terms that dictate the conformational dynamics of such complexes over time. Therefore, we used the MM/GBSA method to predict the overall Free Binding Energy (FBE) between PKa and DON/DRP-104 in comparison to the reference drugs over the entire 200 ns MD simulation period [39, 49]. The BFE of PKa-complexes was −18.58 ± 3.57, −30.62 ± 4.75, −31.41 ± 4.97, and −51.97 ± 3.66 kcal/mol for PKa-DON, PKa-DRP-104, PKa–berotralstat, and PKa–sebetralstat, respectively (Table 3). The results from our MM/GBSA calculations validated the binding affinity of berotralstat, shed light on the advanced inhibitory potential of sebetralstat, and placed DON and DRP-104 in the limelight as potent and novel drug candidates for the treatment of HAE and associated carcinomas. The energy of the van der Waals interactions contributed significantly to the stabilized binding of all inhibitors. On the other hand, the polar solvation energy contributed poorly to the binding of DON, DRP-104, and sebetralstat, whereas the electrostatic energy contributed poorly to the binding of berotralstat.

**Table 3:** Summary of the intermolecular binding energetics of human plasma kallikrein complexes with DON and DRP-104 in relation to the reference drug, berotralstat, and phase 3 inhibitor sebetralstat, expressed in kcal/mol with standard deviations. Binding calculations were performed using the MMGBSA method with 200000 frames

Intermolecular binding energy units (kcal/mol)	Human plasma kallikrein-ligand complex			
	PKa-DON	PKa-DRP-104	PKa–Berotralstat	PKa–Sebetralstat
∆*E*_*vdW*_	−23.27 ± 2.67	−41.44 ± 4.32	−45.42 ± 4.28	−63.87 ± 3.35
∆*E*_*elec*_	−16.48 ± 11.19	8.25 ± 17.69	43.51 ± 29.43	−39.60 ± 8.65
∆*G*_*GB*_	24.68 ± 9.20	7.86 ± 15.57	−23.61 ± 27.01	58.31 ± 7.57
∆*G*_*SA*_	−3.51 ± 0.13	−5.30 ± 0.50	−5.90 ± 0.46	−6.81 ± 0.28
∆*G*_*gas*_	−39.75 ± 10.36	−33.19 ± 17.67	−1.91 ± 29.66	−103.47 ± 9.36
∆*G*_*solv*_	21.17 ± 9.22	2.58 ± 15.44	−29.50 ± 26.83	51.50 ± 7.41
∆*G*_*bind*_	−18.58 ± 3.57	−30.62 ± 4.75	−31.41 ± 4.97	−51.97 ± 3.66

∆*E*_*elec*_ (electrostatic energy), ∆*E*_*vdW*_ (van der Waals energy), ∆*G*_*bind*_ (total free binding energy) ∆*G*_*gas*_ (gas-phase energy), ∆*G*_*GB*_ (polar solvation energy), ∆*G*_*SA*_ (non-polar solvation energy), and ∆*G*_*solv*_ (Total solvation free energy of polar and non-polar states)

### Per-residue energy decomposition (PRED) footprint analysis

The MM/GBSA protocol was used to determine the individual energy contributions of the active site residues to the overall BFE of the various PKa-ligand complexes over the entire 200 ns MD simulation period [31]. PRED analysis factors in two important energy terms: electrostatic and van der Waals. Electrostatic interactions play a crucial role in stabilizing protein structures, mediating interactions between charged residues, and influencing ligand binding. In addition, van der Waals forces contribute to the packing of molecules and stabilization of protein structures by ensuring appropriate intermolecular distances between atoms. The electrostatic energy term significantly surpassed the van der Waals energy term by an 11-fold factor across all the inhibitors, emphasizing its crucial role as an intermolecular force in inhibiting PKa. Notably, the catalytic triad ASP102 contributed the largest electrostatic energy term in the PKa-DRP-104, -berotralstat, and -sebetralstat complexes (Figs. 8C, 9C, and 10C).

**Fig. 7:**
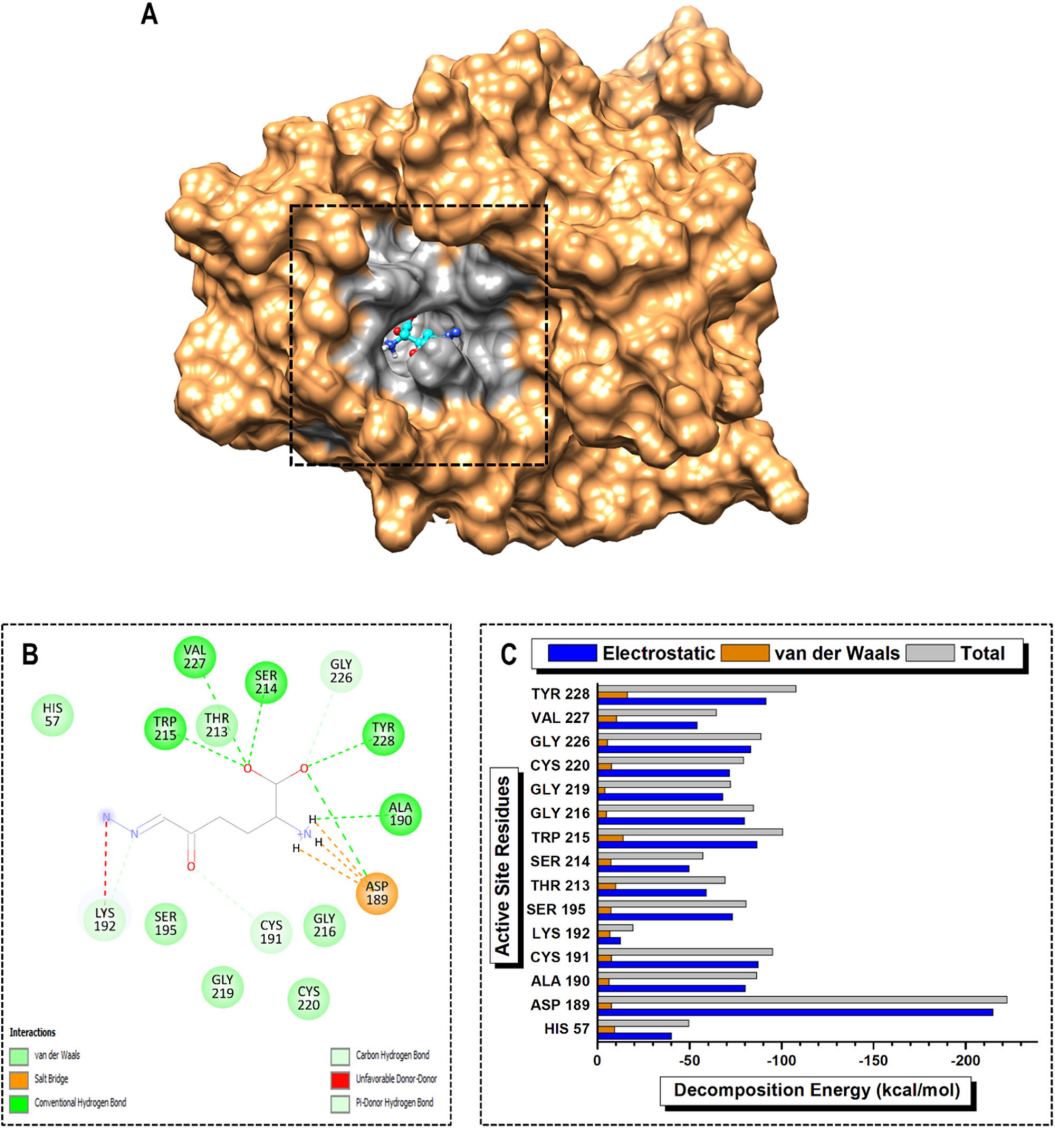
Visual representation of (**A**) protein–ligand complex of PKa (sandy brown) and DON (cyan) and active site residue interaction landmark (light gray), (**B**) 2D protein–ligand interaction mapping, and (**C**) per-residue energy decomposition (PRED) of active site residues over 200 ns MD simulation period

**Fig. 8:**
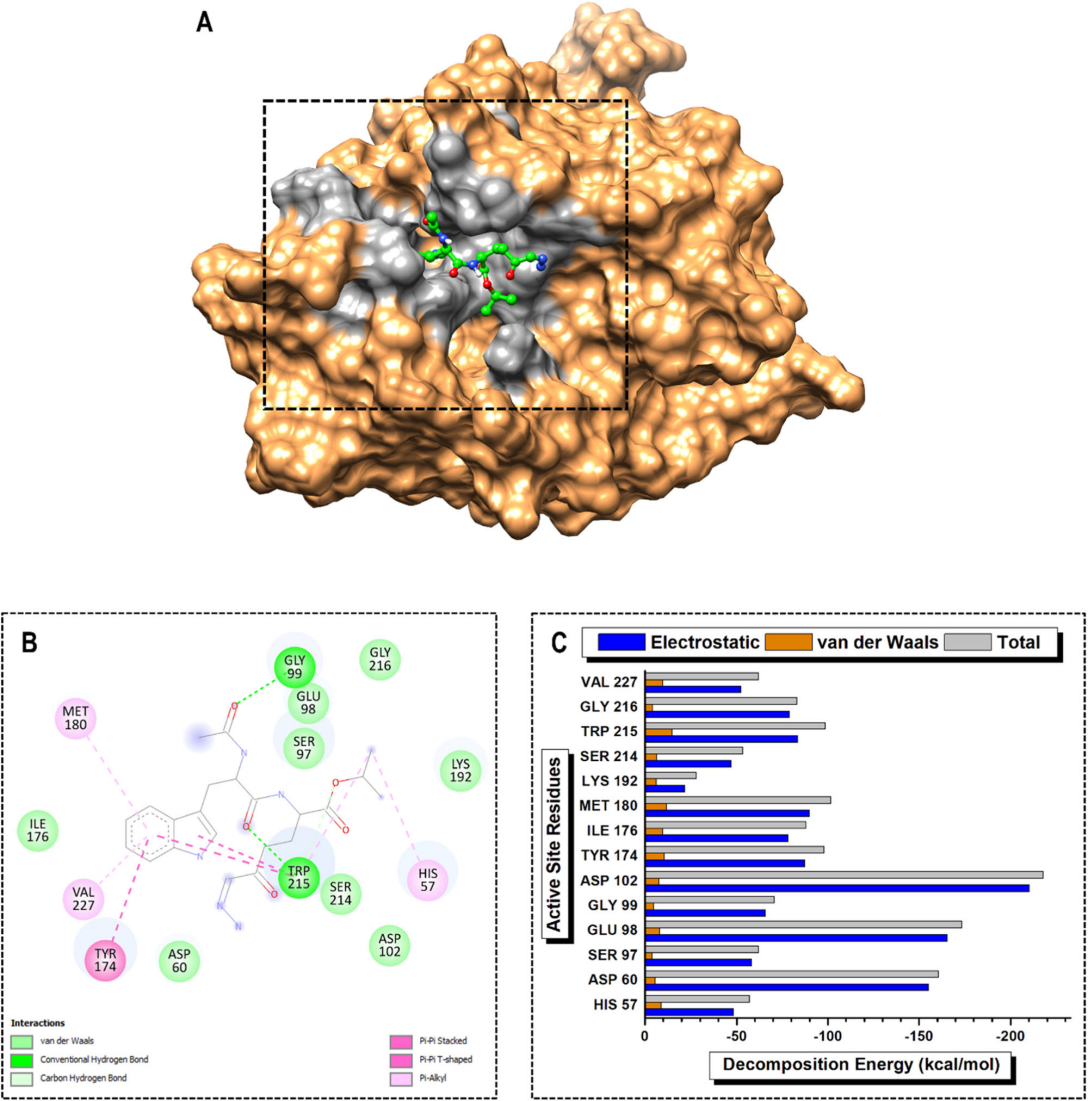
Visual representation of (**A**) protein–ligand complex of PKa (sandy brown) and DRP-104 (green) and active site residue interaction landmark (light gray), (**B**) 2D protein–ligand interaction mapping, and (**C**) per-residue energy decomposition (PRED) of active site residues over 200 ns MD simulation period

In the PKa-DON complex, ASP189, TYR 228, and TRP215 emerged as high-PRED residues, contributing −222.57, −107.80, and −100.64 kcal/mol, respectively. Conversely, SER214, HIS57, and LYS192 were identified as low-PRED residues, with total energy contributions of −57.05, −49.51, and −19.29 kcal/mol, respectively. For smaller contributions to the electrostatic energy term, SER214, HIS57, and LYS192 contributed −49.71, −40.11, and −12.50 kcal/mol, respectively. Additionally, GLY226, GLY216, and GLY219 made poor contributions to the van der Waals energy term at −5.40, −4.93, and −4.13 kcal/mol, respectively (Fig. 7C). In the PKa-DRP-104 complex, the high-PRED residues were ASP102, GLU98, ASP60, and MET180, with total energy contributions of −217.70, −173.43, −160.66, and −101.58 kcal/mol, respectively. In contrast, low-PRED residues included HIS57, SER214, and LYS192, with total energy contributions of −57.23, −53.49, and −27.90 kcal/mol, respectively. SER214 and LYS192 exhibited the lowest electrostatic energy contributions at −46.97 and −21.76 kcal/mol, respectively. Additionally, GLY99, GLY216, and SER97 contributed minimally to the total van der Waals energy term (Fig. 8C).

**Fig. 9:**
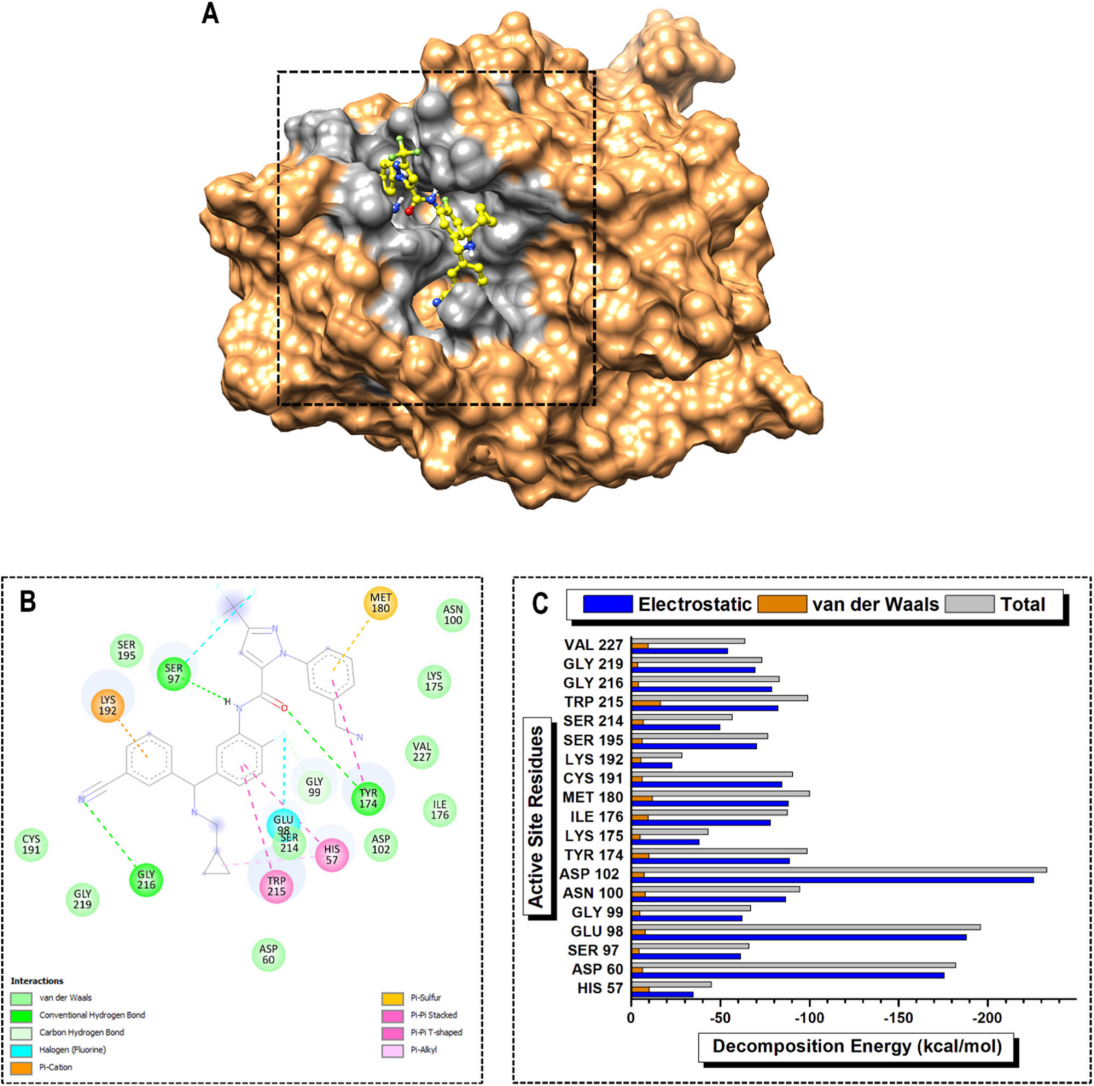
Visual representation of (**A**) protein–ligand complex of PKa (sandy brown) and berotralstat (yellow) and active site residue interaction landmark (light gray), (**B**) 2D protein–ligand interaction mapping, and (**C**) per-residue energy decomposition (PRED) of active site residues over 200 ns MD simulation period

**Fig. 10:**
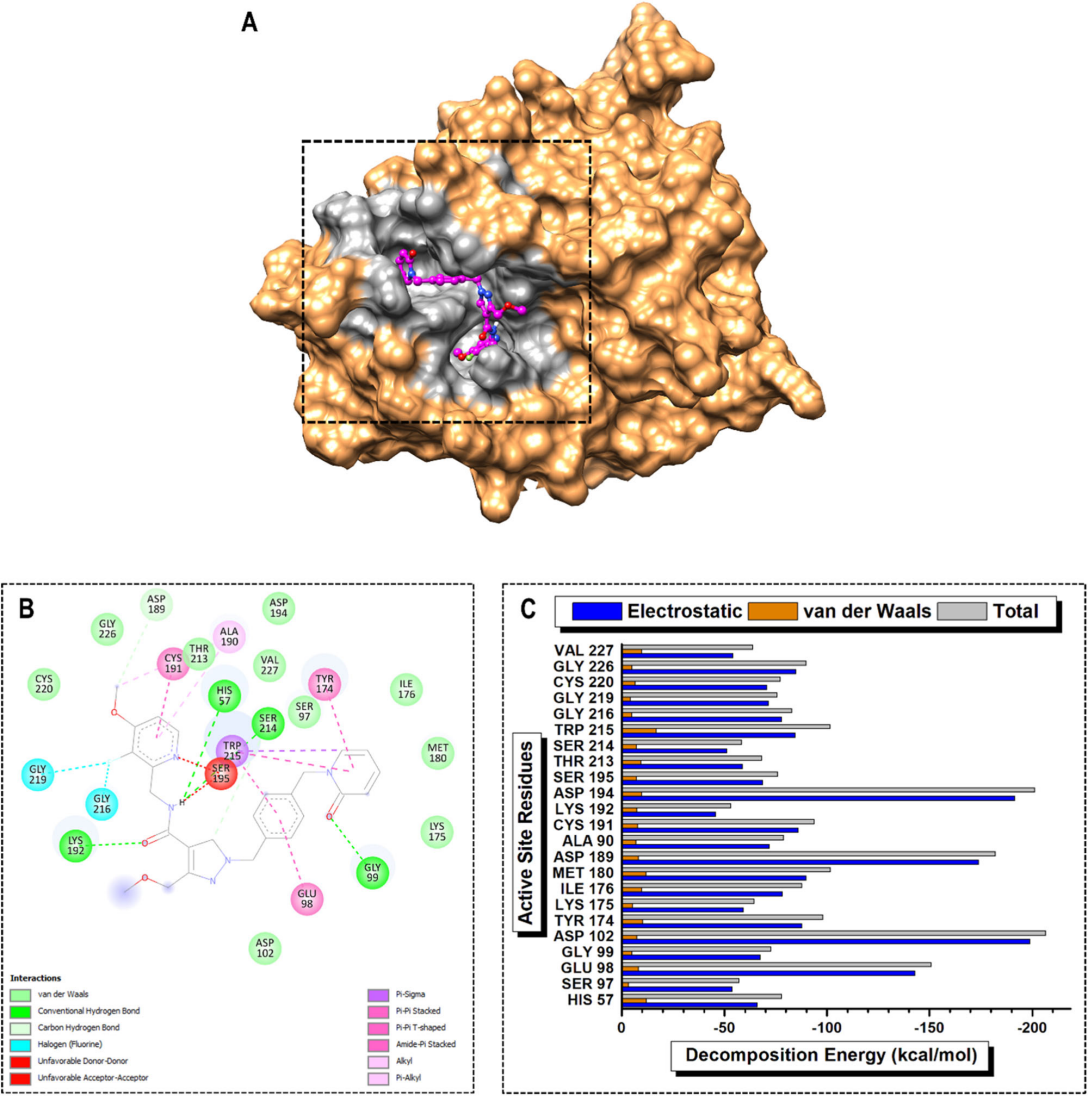
Visual representation of (**A**) protein–ligand complex of PKa (sandy brown) and sebetralstat (magenta) and active site residue interaction landmark (light gray), (**B**) 2D protein–ligand interaction mapping, and (**C**) per-residue energy decomposition (PRED) of active site residues over 200 ns MD simulation period

Furthermore, in the PKa–berotralstat complex, the high-PRED residues were ASP102, GLU98, ASP60, and MET180, with energy contributions of −233.14, −196.14, −182.21, and −100.05 kcal/mol, respectively. In contrast, SER214, HIS57, LYS175, and LYS192 were determined to be low-PRED residues, with individual energy contributions of −56.51, −44.75, −43.05, and −28.38 kcal/mol, respectively. SER214, LYS175, HIS57, and LYS192 contributed the lowest electrostatic energy terms at −49.70, −38.02, −34.59, and −22.89 kcal/mol, respectively. Additionally, GLY99, SER97, GLY216, and GLY219 contributed the lowest van der Waals energy terms at −4.86, −4.55, −4.15, −3.75 kcal/mol, respectively (Fig. 9C). Lastly, in the PKa–sebetralstat complex, the high-PRED residues were ASP102, ASP194, ASP189, and GLU98, with energy contributions of −206.44, −201.32, −182.19, and −150.92 kcal/mol, respectively. Conversely, VAL227, SER214, SER97, and LYS192 were identified as low-PRED residues, with energy contributions of −63.77, −58.25, −56.95, and −53.03 kcal/mol, respectively. The lowest contributions to the total electrostatic energy term were from VAL227, SER97, SER214, and LYS192, with energy terms of −54.15, −53.63, −51.11, and −45.68 kcal/mol, respectively. In addition, GLY99, GLY216, GLY219, and SER97 had the lowest van der Waals energy contributions of −4.95, −4.94, −4.22, and −3.32 kcal/mol. (Fig. 10C). Comparative analysis of electrostatic and van der Waals energy terms across various inhibitors in PKa interactions revealed significant differences in their contributions to binding and stability. This is crucial for elucidating the molecular mechanisms underlying inhibitor interactions with PKa, providing valuable insights for drug design and development.

### High-PRED residue interaction mapping analysis

Protein–ligand interaction mapping analysis was used to further elucidate the functional role of the protein–ligand complex and its potential implications for therapeutic interventions. This will go a long way to guide rational drug design and structure-activity relationship (SAR) studies. In the PKa-DON complex, ASP189 was found to form salt bridges with the ammonia group of the 1,1‐dihydroxypropan‐2‐aminium moiety, while also engaging in a conventional hydrogen bond with 03 of the same group. TYR228 and TRP215 both participated in conventional hydrogen bonds, with TYR228 forming the bond with 03 and TRP215 with 02 of the 1,1‐dihydroxypropan‐2‐aminium moiety. Additionally, CYS191 established a carbon-hydrogen bond with 01 of the acetone group, and GLY226 contributed to the interaction profile by forming a conventional hydrogen bond with 03 of the 1,1‐dihydroxypropan‐2‐aminium moiety (Fig. 7B). In the PKa-DRP-104 complex, ASP102, GLU 98, and ASP60 contributed to the complex formation through van der Waals interactions. MET180 engaged in a π-alkyl interaction with the benzene core of the indole group, whereas TRP215 exhibited π-π stacked interactions with both the benzene and pyrrole core of the indole group. Furthermore, TRP215 formed a conventional hydrogen bond with 03 of the formaldehyde group, a carbon-hydrogen bond with 02 of the propan-2-ol group, and a π-alkyl interaction with C8 of the propan-2-ol group (Fig. 8B). Furthermore, in the PKa–berotralstat complex, ASP102 and ASP60 participated in van der Waals interactions, contributing to the overall stability. GLU98 formed a halogen bond with F1 of the fluorobenzene group. MET 180 engaged in a π-sulfur interaction with the benzene core of the benzylamine group, whereas TRP 215 exhibited a π-π t-shaped interaction with the benzene core of the fluorobenzene group (Fig. 9B). In the PKa–sebetralstat complex, ASP102, ASP194, and MET180 collectively contributed to stability through van der Waals interactions. ASP189 formed a carbon-hydrogen bond with the 2‐ethyl‐3‐fluoro‐4‐methoxypyridine group, whereas GLU98 participated in an amide-π stacked interaction with the benzene core of the 1‐[(4‐methylphenyl) methyl] pyridin‐2‐one group. TRP215 engaged in multiple interactions, including a π-sigma interaction with C2 of the 2(1H)‐pyridinone group and π-π and π-π t-shaped interactions with the benzene core and 2(1H)‐pyridinone core, respectively, of the 1‐[(4‐methylphenyl) methyl] pyridin‐2‐one group (Fig. 10B).

The identified hotspot chemical scaffolds for each complex were the 1,1‐dihydroxypropan‐2‐aminium in the PKa-DON complex, indole and propan-2-ol groups in the PKa-DRP-104 complex, fluorobenzene and benzylamine groups in the PKa–berotralstat complex, and 2‐ethyl‐3‐fluoro‐4‐methoxypyridine and 1‐[(4‐methylphenyl) methyl] pyridin‐2‐one in the PKa–sebetralstat complex. These scaffolds may serve as crucial features for pharmacophore modeling, providing insights into the high-PRED binding characteristics of each complex.

## Conclusion

Our current study aimed to explore the anti-proteasomal therapeutic functions of DON and DRP-104 beyond the scope of chemotherapy and, in this case, as potential novel chemotherapeutic agents against hereditary angioedema (HAE). In addition, while collating in-depth thermodynamic patterns involved in the inhibition of human plasma kallikrein (PKa), this study also probed the inhibitory advantage of Phase 3 S1/S4 sebetralstat over the clinically approved S4 berotralstat. However, berotralstat induced favorable conformational stability with reduced structural flexibility compared with sebetralstat. Comparatively, DON induced favorable structural stability and rigidity, whereas DRP-104 induced restricted residue flexibility in PKa inhibition. Generally, DON induced restricted protein unfolding potential in PKa through increased expulsion of surface loops away from the enclosing solvent and increased formation of intra-atomic hydrogen bonds, compared to DRP-104 and the reference drugs.

Furthermore, the residues were more displaced in the PKa complex with DON than in DRP-104, sebetralstat, and berotralstat. Residue displacements were found to be bidirectional along both principal components. In addition, all regions in PKa underwent strong synchronized residue motion for all inhibitor complexes. DRP-104 and sebetralstat were found to significantly disrupt the SER195-HIS57 and HIS57-ASP102 arrangements, respectively. Finally, PKa formed stable complexes with all inhibitors with favorable free binding energetics. Our study suggests that DON and DRP-104 are potent and novel drug candidates for the treatment of HAE and its associated carcinomas. However, clinical investigations of these candidate drugs remain limited, and should be pursued further using in vitro techniques.

### Supplementary Information

Supplementary Figures

## Data Availability

Data supporting the findings of this study are reported in the paper and are available from the corresponding author upon reasonable request.
