# Supplementary material for: Unveiling therapeutic frontiers: DON/DRP-104 as innovative Plasma kallikrein inhibitors against carcinoma-associated hereditary angioedema shocks - a comprehensive molecular dynamics exploration
Source: Cell Biochem Biophys. 2024 Jun 13;82(2):1159–77. doi: 10.1007/s12013-024-01266-0 (PMC11344713; doi:10.1007/s12013-024-01266-0)
Supplement: Supplementary file 1 — Supplementary Figures [file 12013_2024_1266_MOESM1_ESM.docx]

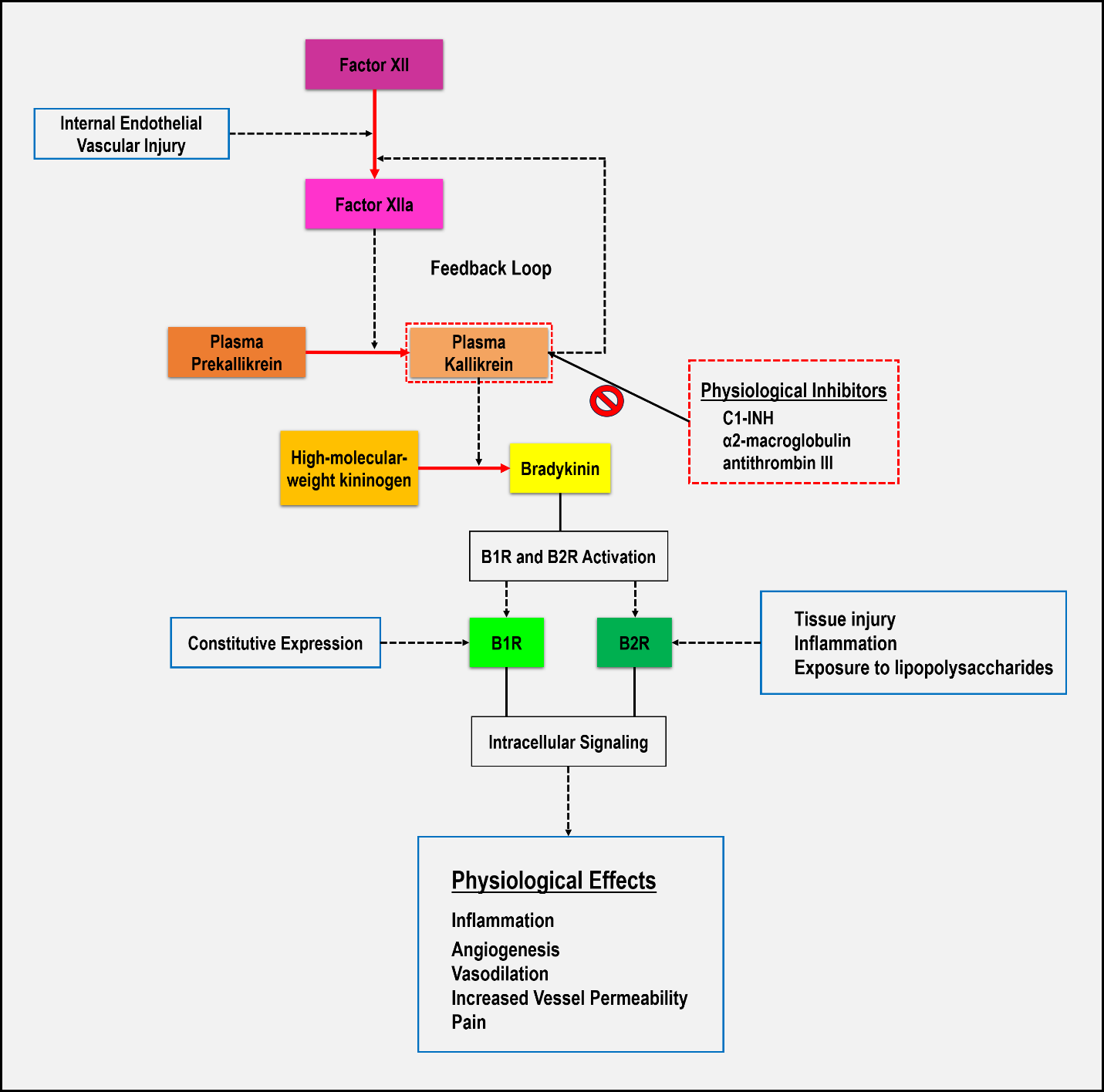


**Fig. 1.** Schematic diagram of the kallikrein-kinin system (KKS) as activated by the intrinsic blood coagulation pathway. The black dashed arrows represent the flow of the kinin cascade, and the solid red arrows represent the activation reactions.


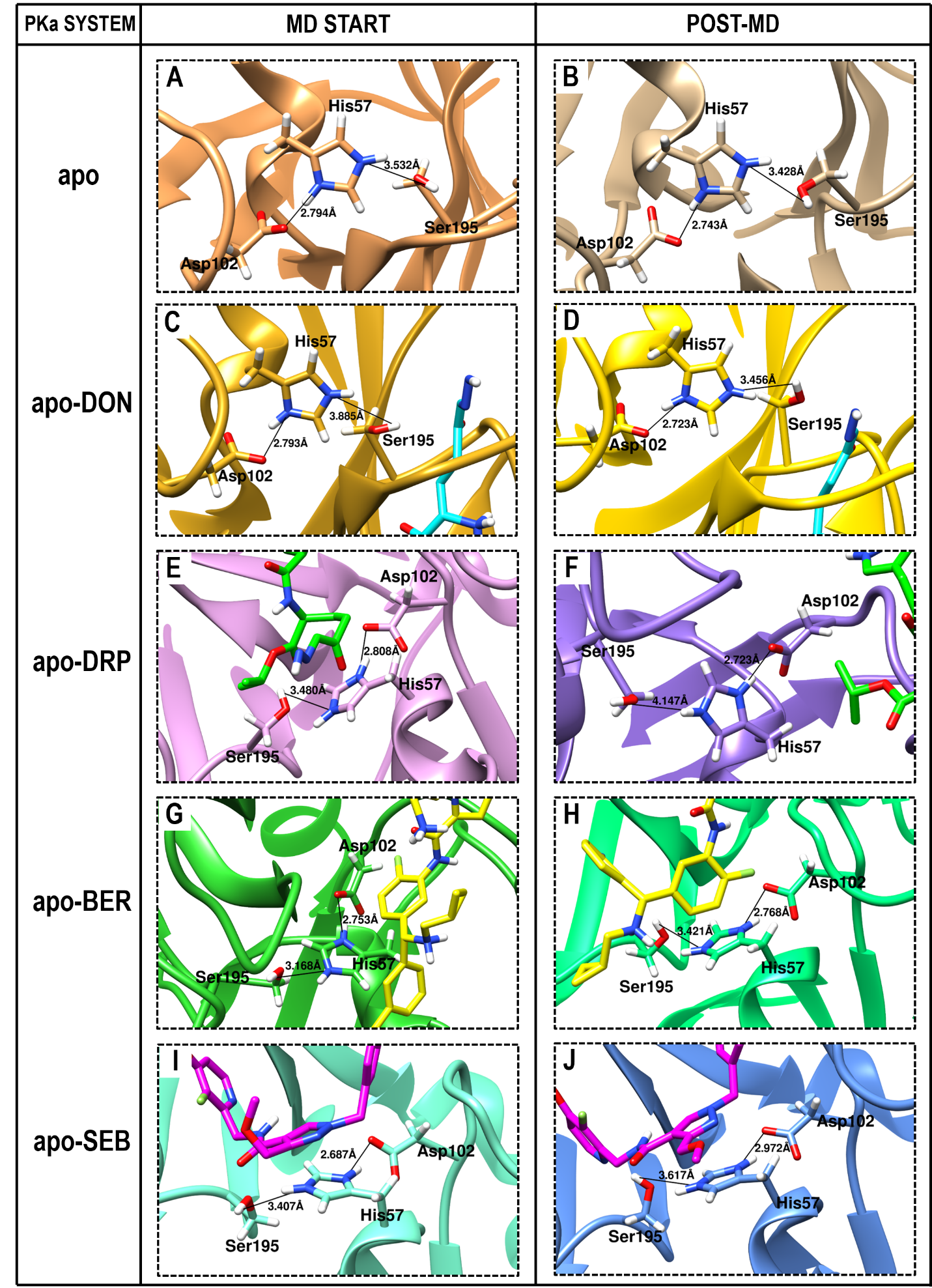


**Fig. 2.** Graphical representation of the comparative pre-/ post-MD catalytic triad distance metric analyses (SER195-HIS57 and HIS57-ASP102) between unligated PKa (apo) **(A)** pre-MD (sandy brown) and **(B)** post-MD (tan), DON- (cyan) **(C)** pre-MD (goldenrod) and **(D)** post-MD (gold), DRP-104- (green) **(E)** pre-MD (plum) and **(F)** post-MD (light purple), berotralstat- (yellow) **(G)** pre-MD (lime green) and **(H)** post-MD (spring green), and sebetralstat- (magenta) **(I)** pre-MD (aquamarine) and **(J)** post-MD (cornflower blue) PKa ligated complex systems.
